# Quantification of Ochratoxin A in 90 spice and herb samples using the ELISA method

**DOI:** 10.25122/jml-2023-0028

**Published:** 2023-09

**Authors:** Ala’ Sirhan, Yazan AlRashdan, Manal Najdawi, Loay Khaled Hassouneh, Ahmad Talhouni, Amjad Abuirmeileh, Qais Jarrar, Rami Ayoub, Lukman Bola Abdulra’uf

**Affiliations:** 1 Department of Pharmacy, Faculty of Pharmacy, Amman Arab University, Amman, Jordan; 2 Department of Applied Pharmaceutical Sciences and Clinical Pharmacy, Faculty of Pharmacy, Isra University, Amman, Jordan; 3 Department of Respiratory Therapy, Faculty of Allied Medical Sciences, Isra University, Amman, Jordan; 4 Department of Chemistry and Industrial Chemistry, Faculty of Pure and Applied Sciences, Kwara State University, Malete, Nigeria

**Keywords:** ELISA, QuEChERS, Mycotoxins, Ochratoxin A, PCA, MRLs: Maximum Residue Levels, OTA: Ochratoxin A, JECFA: Joint Food and Agricultural Organization/World Health Organization Expert Committee on Food Additives, EFSA: European Food Safety Authority, IAC: Immuno-Affinity Column, MIP-SPE: Molecularly Imprinted Polymer Solid Phase Extraction, ELISA: Enzyme-Linked Immunosorbent Assay, LOD: Limit of Detection, LOQ: Limit of Quantification, RSD: Relative Standard Deviation

## Abstract

This study addressed the challenge of accurately detecting mycotoxins in herbs and spices, which have gained popularity as alternative medicines but pose health risks due to potential contamination. We used a competitive direct ELISA kit (Art No. 8610), Veratox for Ochratoxin, to quantify Ochratoxin A in the herb and spice samples. The samples were first prepared using solid-liquid extraction with 70% methanol. The resulting filtrate was then subjected to ELISA analysis. The results of the analysis were then further analyzed using principal component analysis (PCA). In this study, PCA was used to classify the concentration levels of Ochratoxin A based on various factors, such as the packaging type, country of origin, shelf life, and sample weight. The limits of detection (LOD) and quantification (LOQ) values indicate the lowest amount of Ochratoxin A that can be detected and quantified, respectively, with high accuracy and precision. The range of the LOD and LOQ values (0.43-0.58 µg/kg and 1.45-1.95 µg/kg, respectively) suggests that the method used was capable of detecting and quantifying Ochratoxin A in the herb and spice samples at different concentrations with a high degree of accuracy and precision. These results suggest that while most of the samples (73.33%) were below the maximum residue limit (MRL) for Ochratoxin A, a significant number of samples (26.67%) had concentrations of Ochratoxin A that were higher than the MRL. This highlights the importance of monitoring Ochratoxin A in herb and spice samples and ensuring the products are safe for consumption.

## INTRODUCTION

Herbs and spices have long played a crucial role in human existence, improving quality of life through their use as medicinal remedies globally, including in developed countries [1-3]. Although there are differences between herbs and spices, they are often used interchangeably, and definitions for spices often overlap with those for herbs [4]. However, the safety of herbal medicines has been called into question due to their susceptibility to mycotoxin contamination, caused by environmental factors such as high temperatures and heavy rainfall that provide ideal conditions for infestation [4]. Improper drying, high moisture levels, inadequate production methods, poor storage conditions, and lack of farmer awareness can lead to contamination, causing substantial economic losses and posing a significant threat to human health [5-7]. Contaminations can also be introduced during the harvesting and post-harvesting phases of herbs [3]. One of the most common mycotoxins is Ochratoxin A (OTA), produced by several species of *Aspergillus* and *Penicillium fungi* [8]. The contamination of herbs and spices with OTA has become a major concern for human health, as it has been shown to have nephrotoxic, immunotoxic, genotoxic, and carcinogenic effects in animal studies [9, 10]. Additionally, OTA has been classified as a possible human carcinogen by the International Agency for Research on Cancer (IARC) [11]. Agencies, such as the Joint Food and Agricultural Organization/World Health Organization Expert Committee on Food Additives (JECFA), the European Food Safety Authority (EFSA), the United States Environmental Protection Agency, and the International Agency for Research on Cancer monitor and evaluate the potential health risks associated with mycotoxins in food, including herbal products, and set standards for the safe levels of these contaminants. They also provide guidelines for producing, processing and storing herbal products to minimize the risk of mycotoxin contamination. Additionally, they work with manufacturers, growers, and regulators to ensure that herbal products and food are tested for mycotoxins and comply with established standards. Aflatoxins, Ochratoxin, Fumonisins, Zearalenone, and Deoxynivalenol have been identified as the most dangerous mycotoxins regarding their health impact [12]. The presence of mycotoxins at trace levels in herbs makes their detection and accurate measurement a significant challenge. The methods developed for analyzing mycotoxins include immunoassays, chromatography, and spectrometry. However, each method has its limitations and drawbacks, making it difficult to obtain accurate and consistent results [13]. To address this challenge, the use of multiple analytical techniques in combination, such as multi-mycotoxin analysis, can provide more accurate and comprehensive results. Additionally, applying proper sampling and storage methods and good agricultural and manufacturing practices can help minimize the contamination of herbs with mycotoxins [14]. By implementing these measures, we can ensure the safety and quality of herbal products and protect the health of consumers [13]. Over the years, various methods for the extraction of Ochratoxin A from complex samples have been employed, including sonication followed by clean-up using an immuno-affinity column (IAC) [15, 16] or the use of a molecularly imprinted polymer solid phase extraction (MIP-SPE) step for cleaning up herbs. The Quick Easy Cheap Effective Rugged Safe (QuEChERS) technique has also been applied in various modifications for the determination of Ochratoxin A in cereals and cereal products, including *Radix Paeoniae Alba* [17], *Angelica sinensis* [18], and *Silybum marianum* [19]. In addition, liquid chromatography-tandem mass spectrometry (LC-MS/MS) has been widely used as a highly sensitive and specific method for determining mycotoxins in food and herbal products [20, 21]. Other methods like enzyme-linked immunosorbent assay (ELISA), high-performance liquid chromatography (HPLC), and gas chromatography (GC) have also been employed for mycotoxins analysis, but LC-MS/MS is considered the gold standard in mycotoxins analysis [19]. It is essential that appropriate measures are taken to minimize the risk of mycotoxin contamination in herbs and other food products, and the effective analysis of mycotoxins is an important step toward ensuring the safety of food and herbal products for consumers. Using validated and appropriate methods for mycotoxin analysis is crucial in ensuring that the results obtained are reliable and trustworthy. Proper quality control procedures, such as the use of certified reference materials, routine quality control checks, and regular monitoring of the analytical system, should also be implemented in the analysis of mycotoxins [22]. The ELISA method has been widely used to determine Ochratoxin A in food and herbal samples due to its specificity and sensitivity [21]. In this method, a monoclonal or polyclonal antibody is used to recognize the Ochratoxin A molecules in the sample, and the reaction between the antibody and the mycotoxin is then quantified using a detection reagent. The ELISA method provides accurate and precise results and has been validated to determine Ochratoxin A in different food matrices [22]. However, the ELISA method has some limitations, such as the monoclonal or polyclonal antibody cost and the need for specialized equipment and trained personnel. The ELISA method may also not be suitable for determining other mycotoxins, as a different antibody is needed for each mycotoxin [23]. To overcome these limitations, other methods, such as liquid chromatography-mass spectrometry (LC-MS) and high-performance liquid chromatography (HPLC), have also been applied for the determination of mycotoxins in food and herbal samples [20]. It is important to monitor the presence of mycotoxins in herbal products to guarantee public health and ensure the efficacy and safety of these products. The ELISA method has been widely used to determine Ochratoxin A in herbal samples, but it has some limitations. Therefore, a combination of different methods should be used to determine the presence of mycotoxins in herbal products to ensure accurate and reliable results. This study aimed to determine Ochratoxin A in herb and spice samples using a competitive direct ELISA kit (Art No. 8610), with Veratox for Ochratoxin. Additionally, principal component analysis (PCA) was employed to explore the relationships between Ochratoxin A concentration and various factors, including sample type, packaging, source of origin, shelf life, and sample weight.

## MATERIAL AND METHODS

### Sample preparation

Ten grams of each of the 90 spice or herb samples were dissolved in 100 milliliters of double-distilled water, heated to 50°C, and stirred using a hot plate stirrer. The sample was then centrifuged at 3500 g for 10 minutes at 10°C. The upper fat layer was removed, and the herb serum was directly analyzed for Ochratoxin A (OTA) using a specific ELISA kit. To prepare the herb samples, 50 grams of the ground and homogenized herb was blended with 250 milliliters of 70% methanol for 1 minute in a high-speed blender. Sample recovery was done with 50 grams of non-contaminated herb samples with three different fortification levels, with OTA standard spiked at 5.0, 10.0, and 20.0 micrograms per kilogram of herb. The spiked samples were left overnight for 14 hours in the dark at room temperature (with humidity between 30% and 60%) to allow solvent evaporation and for PDE5 inhibitors to be absorbed into the matrix. The samples were then extracted using the following steps: the extract was filtered by passing a minimum of 5 milliliters through a Whatman #1 filter (or a Neogen filter syringe), and the resulting filtrate was collected as a sample, prepared for subsequent ELISA analysis.

### Analysis of OTA by competitive enzyme-linked immunosorbent assay (ELISA)

Ochratoxin A (OTA) was determined using Veratox for Ochratoxin ELISA kit from Neogen Corporation with a detection limit of 1 ppt. The kit was kept at room temperature (20-25°C) for an hour before use. The standard curve was prepared using the OTA standard solutions of 0, 2, 5, 10, and 25 ppb.

Following the preparation steps, sample analysis was conducted in accordance with the manufacturer's instructions. To initiate the analysis, 100 µL of conjugate was added to each designated, red-marked mixing well. Subsequently, 100 µL of both controls and samples were added to their respective wells. After mixing, 100 µL was transferred to the antibody wells. Excess liquid from the antibody wells was then carefully discarded. To ensure accuracy, the wells underwent five washes with deionized water, and any remaining water was removed by gently tapping the wells on a paper towel. The next step involved transferring 100 µL of substrate from the reagent boat to the antibody wells using a 12-channel pipettor, followed by an incubation period of 10 minutes. Finally, 100 µL of Red Stop from the reagent boat was added to the antibody wells. The absorbance was measured at 650 nm using a microwell reader with a 650 nm filter. The OTA sheet supplied with the kit was used to generate a standard curve and calculate the OTA concentration in the samples.

### Method validation

This method was validated in-house for linearity, accuracy, intra- and inter-day precisions, limit of detection (LOD), and limit of quantification (LOQ). Linearity was confirmed by testing a standard Ochratoxin A (OTA) solution in the concentration range of 2.0 to 25 µg/L. Calibration was carried out using the standard solution, and quantification was performed using the least square method. Statistical comparison of the mean was conducted using an ANOVA test (p<0.05). Accuracy was assessed by determining the recoveries of OTA in various samples, including clean thyme leaves, rosemary, and mixed spices samples spiked with OTA standard at levels of 5.0, 10.0, and 20.0 µg/kg, and analyzed in triplicate.

The sensitivity of the method was evaluated by determining the limit of detection (LOD) and the limit of quantification (LOQ). LOD was established as the lowest concentration that produced a response three times higher than the baseline noise, while LOQ was defined as the concentration resulting in a response ten times higher than the baseline noise.

The precision of the method for detecting Ochratoxin A (OTA) in samples was rigorously assessed, both for intra-day and inter-day variations. The intra-day precision was evaluated by assaying five replicates of the same sample at a spiked level of 10 µg/L OTA on the same day, while the inter-day precision was evaluated by analyzing five replicates of the same sample at a spiked level of 10 µg/L OTA over three consecutive days.

## RESULTS

The concentration range tested demonstrated strong linear relationships with correlation coefficients of 0.997 for the targeted analyte. As shown in Table 1, the recoveries ranged from 90.2±1.0% to 99.6±5.5%, with a relative standard deviation (RSD) ranging from 1.0 to 6.0% when analyzing samples spiked with 5, 10, and 20 µg/kg. These recovery results and relative standard deviation were considered satisfactory according to the European Commission decision 2002/657/EC [21].

**Table 1:** Recovery, LOD, LOQ, intra- and inter-day precision of OTA

Matrix	Recovery (%)^a^			LOD µg/kg	LOQ µg/kg	Intra-day %	Inter-day %
	5 µg/kg	10 µg/kg	20 µg/kg				
Thyme leaves	99.6±2.4	90.8±5.6	95.2±1.3	0.56	1.87	1.2	2.5
Rosemary	99.3±3.3	92.1±3.4	95.6±5.5	0.58	1.95	1.7	2.4
Mixed spices	98.0±3.4	98.0±6.0	90.2±1.0	0.43	1.45	2.0	3.1

a : Recovery ± RSD (%) (n=5)

The LOQ ranged from 1.45-1.95 µg/kg, while the LOD ranged from 0.43-0.58 µg/kg (as shown in Table 1).

The intra-day precision was between 1.2 and 2.0, while the inter-day precision was between 2.4 and 3.1%. These values, below the acceptable maximum, demonstrate the reproducibility and repeatability of the method, which can be considered selective, precise, and robust.

### Analysis of real samples

Samples were analyzed for the presence of Ochratoxin A, and the samples were obtained from the Jordanian market with origins from different countries, as indicated in Table 2.

**Table 2:** The 90 samples analyzed

Country	No.
Brazil	1
China	1
Egypt	6
India	3
Indonesia	1
Jordan	51
Lebanon	3
Mexico	1
Nigeria	1
Pakistan	2
Sudan	2
United State	9
Four samples could not be traced	9
Total	90

The spice and herb samples were packed in different package types, such as boxes (17), glass jars (7), and plastic bags (66).

**Figure 1:**
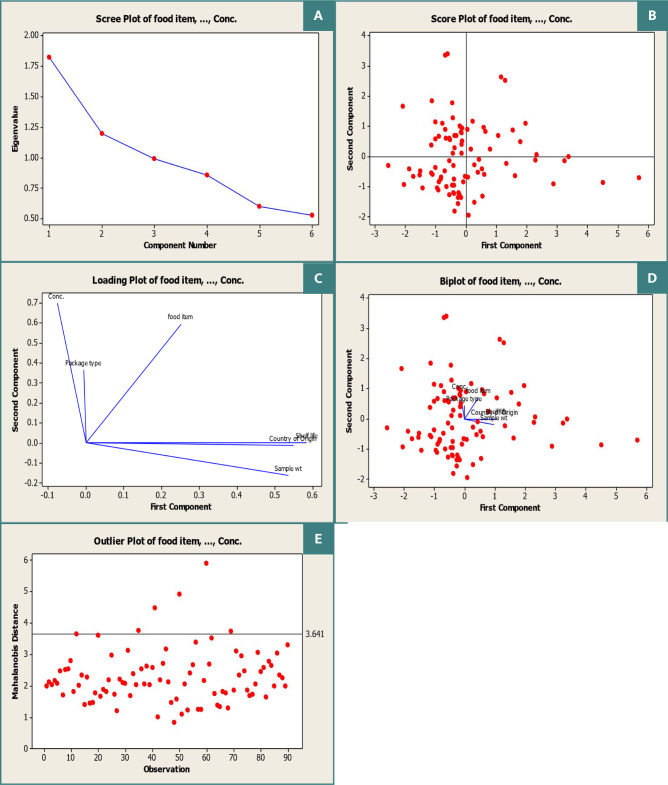
Principal component analysis (PCA) of Ochratoxin A in spices and herbs showing (A) scree, (B) score, (C) loading, (D) biplot, and (E) outlier plots.

The detection of Ochratoxin A in herbs and spices is crucial due to the unique conditions of their production and storage. Of the 90 samples analyzed, Ochratoxin A was present in varying concentrations, with 73.33% (66 samples) being below the maximum residue levels set by Regulation 1881/2006 of the European Commission (Table 3). However, 26.67% (23 samples) had concentrations exceeding the maximum residue levels. Most of the samples with lower concentrations (15-20 µg/kg) were locally produced in Jordan, with some imported from countries such as Pakistan, Egypt, Syria, Sudan, and the United States. The highest concentration of Ochratoxin A found was 166.82 µg/kg in ground spices produced in Jordan. The results of the principal component analysis, as seen in Figure 1(A-E) and Table 4, indicated that the first component had a significant positive effect on the origin, shelf life, and weight of the samples. In contrast, the second component showed a strong positive correlation with the type of spice and the concentration of Ochratoxin A in each sample. The PCA results showed that shelf-life accounted for 58.40% of the total variance in the first principal component, reflecting the length and type of storage, while the country of origin accounted for 55.00% of the observed variance, potentially due to environmental and climatic conditions in the country of production. The weight of the sample accounted for 53.60% of the total variance. The loading plot revealed that both concentration and packaging type had positive effects on Ochratoxin A levels, while the type of spices, shelf-life, sample weight, and country of origin all had positive effects as well.

**Table 3:** Ochratoxin A contaminations in spice samples (Total Ochratoxin A (µg/kg))

Sample	Ochratoxin A (µg/kg)	Sample	Ochratoxin A (µg/kg)	Sample	Ochratoxin A (µg/kg)
Abido special spices	6.216	Gory roses	14.44	Mixed spices	1.84
Alexandrian Senna	3.77	Grain spices	5.46	Mixed spices	6.88
All spice	6.09	Green tea	42.37	Moringa	45.84
Anise grain	1.56	Ground biryani spices	8.40	Moringa	16.12
Badia dill	5.93	Ground cinnamon	81.13	Moringa	30.88
Black pepper	38.25	Ground cinnamon	101.89	Nettles	9.06
Boiled chicken and meat mixture spices	7.30	Ground curry	0.71	Orijano	41.39
Bukhari spices	2.70	Ground garlic	117.65	Paprika spices	16.55
Caesalpiniaceous	6.20	Ground ginger	1.51	Potato sprinkle spices	164.06
Cajun seasoning	1.67	Ground nutmeg spice	1.89	Red pepper	35.86
Caraway	6.53	Ground spices	166.82	Rose flower	130.80
Carnation flower	138.40	Ground turmeric	2.67	Roselle	17.51
Chamomile	5.768	Ground white pepper	2.29	Roselle	117.65
Chamomile	12.39	Grounded sumac	20.90	Rosemary	0.80
Chicken shawarma spices	9.06	Grounded sweet red pepper	0.65	Rosemary	2.84
Chicken shawarma spices	1.96	Grounded sweet red pepper	33.22	Rosemary	31.17
Chicken shawarma spices	13.68	Guava leaves	21.38	Sage	6.04
Chinese spices	7.12	Hamburger spice	32.89	Sage	4.98
Circaea	0.96	Hawthorn	6.93	Sage	23.20
onion and hot pepper mixture	5.87	Himalayan pink salt spices	0.69	Seven spices	37.43
Curry	9.29	Italian herbs	2.93	Soft cinnamon	61.24
Dried basil	2.57	Kabsa spices	16.44	Soft kibbe spice	18.89
Dry mint	7.82	Kudra spices	4.10	Spices lemon, salt and turmeric	5.10
Dry mint	5.32	Laureate paper	13.97	Sweet paprika	2.83
Fajita seasoning	2.62	Laureate paper	3.42	Tandoori spices	3.72
Fenugreek beans	9.29	Lemon and dill seasoning for fish	2.33	Thyme leaves	5.51
Fish seasoning	5.03	Marjoram	3.03	Thyme leaves	3.36
Fish spices	1.82	Marjoram	49.82	Thyme leaves	3.42
Fish spices	1.29	Meat shawarma spices	7.49	Upside down spice	7.40
Ghee and mansaf spices	10.27	Mixed chicken spices	5.74	Yarrows	138.40

**Table 4:** Eigenanalysis of the correlation matrix

Variable	PC1	PC2	PC3	PC4	PC5	PC6
Spice	0.251	0.594	-0.402	-0.432	0.478	0.086
Package type	-0.006	0.364	0.867	-0.328	-0.001	-0.088
Origin	0.550	-0.013	0.867	-0.339	-0.645	-0.376
Shelf life	0.584	0.002	0.165	0.226	-0.101	0.755
Weight	0.536	-0.163	0.169	0.389	0.506	-0.500
Conc. level	-0.076	0.698	-0.074	0.623	-0.300	-0.153

## DISCUSSION

The results obtained in this study underscore the critical importance of regularly analyzing food items for quality control and contaminant inspection. The necessity of determining the presence of Ochratoxins A and other mycotoxins in spices is due to their inherent toxicity, posing potential health risks that may not manifest immediately but can accumulate in the human body over time. The result obtained in this study align with the studies of El-Dawy *et al*. [24], Taneinova *et al*. [25], Omotayo *et al*. [26], and Nguegwouo *et al*. [27] which detected Ochratoxin A in ginger obtained from Egypt, Nigeria, South Africa and Cameroon, respectively, at concentrations of 6.7 µg/kg, 0.2 µg/kg, 3.6-411.1 µg/kg, and 1.91 µg/kg, respectively. Additionally, our results align with the findings of Zaied *et al*. [28], who employed ELISA methods to detect Ochratoxin A concentrations ranging from 203 to 290 µg/kg in caraway, coriander, curcumin, black pepper, and red pepper samples obtained in Tunisia. There was a relationship between the concentration of Ochratoxin A found in herb and spice samples and the country of origin, shelf life, and weight of the samples.

## CONCLUSION

Promoting good agricultural practices throughout the production process of herbs and spices is important to ensure their quality, safety, and compliance with international regulations. The widespread use of spices and herbs for health and well-being highlights the need for careful monitoring of mycotoxin contamination, especially for Ochratoxin A, which is highly toxic and has been a global concern. In this study, the presence of Ochratoxin A was determined in 90 herb and spice samples using an enzyme-linked immunosorbent assay (ELISA), and all the samples were found to contain Ochratoxin A at different concentrations. The contamination level was dependent on the country of origin and packaging methods, which could be attributed to environmental and climatic factors. In conclusion, the results of this study highlight the importance of monitoring the presence of Ochratoxin A in herb and spice samples and the need to implement appropriate measures to reduce its contamination. Using ELISA in conjunction with PCA analysis proved to be an effective method for determining and classifying Ochratoxin A in herb and spice samples. Further research is needed to determine the long-term health effects of consuming contaminated herbs and spices and to develop measures to minimize the risks associated with their consumption.

## References

[1] Wakhungu CN, Okoth S, Wachira P, Otieno NA (2021). Mycotoxins contaminating herbs and spices in Africa: A review. Afr J Biol Sci.

[2] Ałtyn I, Twarużek M (2020). Mycotoxin Contamination Concerns of Herbs and Medicinal Plants. Toxins (Basel).

[3] Pallarés N, Berrada H, Font G, Ferrer E (2022). Mycotoxins occurrence in medicinal herbs dietary supplements and exposure assessment. J Food Sci Technol.

[4] Pickova D, Toman J, Ostry V, Malir F (2021). Natural Occurrence of Ochratoxin A in Spices Marketed in the Czech Republic during 2019-2020. Foods.

[5] Cho SH, Lee CH, Jang MR, Son YW (2008). Aflatoxins contamination in spices and processed spice products commercialized in Korea. Food Chem.

[6] Ekhtelat M, Badpa F, Khorasgani ZN, Azemi E (2018). Enzyme-linked Immunosorbent Assay and High-performance Liquid Chromatography analysis of Ochratoxin A in Zataria multifori and Foeniculum vulgare in Ahvaz (Iran). Asian J Pharm.

[7] IARC (2012). Economics of mycotoxins: evaluating costs to society and cost-effectiveness of interventions. IARC Science Publication.

[8] van Egmond HP, Schothorst RC, Jonker MA (2007). Regulations relating to mycotoxins in food: perspectives in a global and European context. Anal Bioanal Chem.

[9] Ostry V (2008). Alternaria mycotoxins: An overview of chemical characterization, producers, toxicity, analysis and occurrence in foodstuffs. World Mycotoxin.

[10] Pitt JI, Morris J. G., Potter M. E. (2013). Chapter 30 - Mycotoxins. Foodborne Infections and Intoxications (Fourth Edition).

[11] Armendáriz CR, Fernández ÁJG, Gironés MCLR, de la Torre AH, Wexler P. (2014). Mycotoxins. Encyclopedia of Toxicology (Third Edition).

[12] (2002). European Union 2002/657/EC: Commission Decision of 12 August 2002 implementing Council Directive 96/23/EC concerning the performance of analytical methods and the interpretation of results (Text with EEA relevance) (notified under document number C (2002), 3044). Official Journal of European Union.

[13] Zhang L, Dou X, Zhang C, Logrieco AE, Yang M (2018). A review of current methods for analysis of mycotoxins in herbal medicines. Toxins.

[14] Whitaker TB (2003). Standardisation of mycotoxin sampling procedures: an urgent necessity. Food Control.

[15] Kong WJ, Liu SY, Qiu F, Xiao XH, Yang MH (2013). Simultaneous multi-mycotoxin determination in nutmeg by ultrasound-assisted solid-liquid extraction and immunoaffinity column clean-up coupled with liquid chromatography and on-line post-column photochemical derivatization-fluorescence detection. Analyst.

[16] Wen J, Kong W, Wang J, Yang M (2013). Simultaneous determination of four aflatoxins and ochratoxin A in ginger and related products by HPLC with fluorescence detection after immunoaffinity column clean-up and postcolumn photochemical derivatization. J Sep Sci.

[17] Sirhan AY, Tan GH, Wong RCS (2012). QuEChERS Extraction and HPLC-FLD Determination of Ochratoxin A in Cereals and Cereal Products. Asian J Chem.

[18] Xing Y, Meng W, Sun W, Li D (2016). Simultaneous qualitative and quantitative analysis of 21 mycotoxins in Radix Paeoniae Alba by ultra-high performance liquid chromatography quadrupole linear ion trap mass spectrometry and QuEChERS for sample preparation. J Chromatogr B Analyt Technol Biomed Life Sci.

[19] Liu Q, Kong W, Guo W, Yang M (2015). Multi-class mycotoxins analysis in Angelica sinensis by ultra fast liquid chromatography coupled with tandem mass spectrometry. J Chromatogr B Analyt Technol Biomed Life Sci.

[20] Arroyo-Manzanares N, García-Campaña AM, Gámiz-Gracia L (2013). Multiclass mycotoxin analysis in Silybum marianum by ultra high performance liquid chromatography-tandem mass spectrometry using a procedure based on QuEChERS and dispersive liquid-liquid microextraction. J Chromatogr A.

[21] Castegnaro M, Tozlovanu M, Wild C, Molinié A (2006). Advantages and drawbacks of immunoaffinity columns in analysis of mycotoxins in food. Mol Nutr Food Res.

[22] European Union (2004). DIRECTIVE 2004/24/EC OF THE EUROPEAN PARLIAMENT AND OF THE COUNCIL of 31 March 2004 amending, as regards traditional herbal medicinal products, Directive 2001/83/EC on the Community code relating to medicinal products for human use. Official Journal of the European Union.

[23] Meulenberg EP (2012). Immunochemical methods for ochratoxin A detection: a review. Toxins (Basel).

[24] El-Dawy EGAE, Yassein AS, El-Said AH (2019). Detection of mycobiota, aflatoxigenic and ochratoxigenic genes, and cytotoxic ability in spices. Food Sci Nutr.

[25] Tančinová D, Mokrý M, Barboráková Z, Mašková Z (2014). Mycobiota of spices and aromatic herbs. Potravinarstvo.

[26] Omotayo OP, Omotayo AO, Mwanza M, Babalola OO (2019). Prevalence of Mycotoxins and Their Consequences on Human Health. Toxicol Res.

[27] Nguegwouo E, Sone LE, Tchuenchieu A, Tene HM (2018). Ochratoxin A in black pepper, white pepper and clove sold in Yaoundé (Cameroon) markets: contamination levels and consumers’ practices increasing health risk. Int J Food Contam.

[28] Zaied C, Abid S, Bouaziz C, Chouchane S (2010). Ochratoxin A levels in spices and dried nuts consumed in Tunisia. Food Addit Contam Part B Surveill.

